# Supplementary material for: An open-source framework for large-scale, flexible evaluation of biomedical text mining systems
Source: J Biomed Discov Collab. 2008 Jan 29;3:1. doi: 10.1186/1747-5333-3-1 (PMC2276192; doi:10.1186/1747-5333-3-1)
Supplement: Additional file 1 — Evaluation platform source code. The additional file contains the software evaluation framework discussed in this paper. The most current release can be downloaded from SourceForge.net [1]. See the accompanying README file for instructions on installation and use. Also included in the distribution is a collection of UIMA wrappers for some commonly used BioNLP tools and annotated corpora. [file 1747-5333-3-1-S1.zip › bionlp_uima_repository-1.0.1/docs/api/src-html/edu/uchsc/ccp/uima/annotation/CCPDocumentInformation_Type.html]

```
001    
002    /* First created by JCasGen Tue Aug 28 16:44:10 MDT 2007 */
003    package edu.uchsc.ccp.uima.annotation;
004    
005    import org.apache.uima.cas.Feature;
006    import org.apache.uima.cas.FeatureStructure;
007    import org.apache.uima.cas.Type;
008    import org.apache.uima.cas.impl.CASImpl;
009    import org.apache.uima.cas.impl.FSGenerator;
010    import org.apache.uima.cas.impl.FeatureImpl;
011    import org.apache.uima.cas.impl.TypeImpl;
012    import org.apache.uima.jcas.JCas;
013    import org.apache.uima.jcas.JCasRegistry;
014    
015    /** The CCPDocumentInformation annotation includes document metadata such as the document ID, document collection ID, secondary document IDs, document size, etc.
016     * Updated by JCasGen Tue Aug 28 16:44:10 MDT 2007
017     * @generated */
018    public class CCPDocumentInformation_Type extends CCPDocumentAnnotation_Type {
019      /** @generated */
020      protected FSGenerator getFSGenerator() {return fsGenerator;}
021      /** @generated */
022      private final FSGenerator fsGenerator = 
023        new FSGenerator() {
024          public FeatureStructure createFS(int addr, CASImpl cas) {
025                             if (CCPDocumentInformation_Type.this.useExistingInstance) {
026                               // Return eq fs instance if already created
027                         FeatureStructure fs = CCPDocumentInformation_Type.this.jcas.getJfsFromCaddr(addr);
028                         if (null == fs) {
029                           fs = new CCPDocumentInformation(addr, CCPDocumentInformation_Type.this);
030                               CCPDocumentInformation_Type.this.jcas.putJfsFromCaddr(addr, fs);
031                               return fs;
032                         }
033                         return fs;
034            } else return new CCPDocumentInformation(addr, CCPDocumentInformation_Type.this);
035              }
036        };
037      /** @generated */
038      public final static int typeIndexID = CCPDocumentInformation.typeIndexID;
039      /** @generated 
040         @modifiable */
041      public final static boolean featOkTst = JCasRegistry.getFeatOkTst("edu.uchsc.ccp.uima.annotation.CCPDocumentInformation");
042     
043      /** @generated */
044      final Feature casFeat_documentID;
045      /** @generated */
046      final int     casFeatCode_documentID;
047      /** @generated */ 
048      public String getDocumentID(int addr) {
049            if (featOkTst && casFeat_documentID == null)
050          jcas.throwFeatMissing("documentID", "edu.uchsc.ccp.uima.annotation.CCPDocumentInformation");
051        return ll_cas.ll_getStringValue(addr, casFeatCode_documentID);
052      }
053      /** @generated */    
054      public void setDocumentID(int addr, String v) {
055            if (featOkTst && casFeat_documentID == null)
056          jcas.throwFeatMissing("documentID", "edu.uchsc.ccp.uima.annotation.CCPDocumentInformation");
057        ll_cas.ll_setStringValue(addr, casFeatCode_documentID, v);}
058        
059      
060     
061      /** @generated */
062      final Feature casFeat_documentCollectionID;
063      /** @generated */
064      final int     casFeatCode_documentCollectionID;
065      /** @generated */ 
066      public int getDocumentCollectionID(int addr) {
067            if (featOkTst && casFeat_documentCollectionID == null)
068          jcas.throwFeatMissing("documentCollectionID", "edu.uchsc.ccp.uima.annotation.CCPDocumentInformation");
069        return ll_cas.ll_getIntValue(addr, casFeatCode_documentCollectionID);
070      }
071      /** @generated */    
072      public void setDocumentCollectionID(int addr, int v) {
073            if (featOkTst && casFeat_documentCollectionID == null)
074          jcas.throwFeatMissing("documentCollectionID", "edu.uchsc.ccp.uima.annotation.CCPDocumentInformation");
075        ll_cas.ll_setIntValue(addr, casFeatCode_documentCollectionID, v);}
076        
077      
078     
079      /** @generated */
080      final Feature casFeat_documentSize;
081      /** @generated */
082      final int     casFeatCode_documentSize;
083      /** @generated */ 
084      public int getDocumentSize(int addr) {
085            if (featOkTst && casFeat_documentSize == null)
086          jcas.throwFeatMissing("documentSize", "edu.uchsc.ccp.uima.annotation.CCPDocumentInformation");
087        return ll_cas.ll_getIntValue(addr, casFeatCode_documentSize);
088      }
089      /** @generated */    
090      public void setDocumentSize(int addr, int v) {
091            if (featOkTst && casFeat_documentSize == null)
092          jcas.throwFeatMissing("documentSize", "edu.uchsc.ccp.uima.annotation.CCPDocumentInformation");
093        ll_cas.ll_setIntValue(addr, casFeatCode_documentSize, v);}
094        
095      
096     
097      /** @generated */
098      final Feature casFeat_secondaryDocumentIDs;
099      /** @generated */
100      final int     casFeatCode_secondaryDocumentIDs;
101      /** @generated */ 
102      public int getSecondaryDocumentIDs(int addr) {
103            if (featOkTst && casFeat_secondaryDocumentIDs == null)
104          jcas.throwFeatMissing("secondaryDocumentIDs", "edu.uchsc.ccp.uima.annotation.CCPDocumentInformation");
105        return ll_cas.ll_getRefValue(addr, casFeatCode_secondaryDocumentIDs);
106      }
107      /** @generated */    
108      public void setSecondaryDocumentIDs(int addr, int v) {
109            if (featOkTst && casFeat_secondaryDocumentIDs == null)
110          jcas.throwFeatMissing("secondaryDocumentIDs", "edu.uchsc.ccp.uima.annotation.CCPDocumentInformation");
111        ll_cas.ll_setRefValue(addr, casFeatCode_secondaryDocumentIDs, v);}
112        
113       /** @generated */
114      public String getSecondaryDocumentIDs(int addr, int i) {
115            if (featOkTst && casFeat_secondaryDocumentIDs == null)
116          jcas.throwFeatMissing("secondaryDocumentIDs", "edu.uchsc.ccp.uima.annotation.CCPDocumentInformation");
117        if (lowLevelTypeChecks)
118          return ll_cas.ll_getStringArrayValue(ll_cas.ll_getRefValue(addr, casFeatCode_secondaryDocumentIDs), i, true);
119        jcas.checkArrayBounds(ll_cas.ll_getRefValue(addr, casFeatCode_secondaryDocumentIDs), i);
120            return ll_cas.ll_getStringArrayValue(ll_cas.ll_getRefValue(addr, casFeatCode_secondaryDocumentIDs), i);
121      }
122       
123      /** @generated */ 
124      public void setSecondaryDocumentIDs(int addr, int i, String v) {
125            if (featOkTst && casFeat_secondaryDocumentIDs == null)
126          jcas.throwFeatMissing("secondaryDocumentIDs", "edu.uchsc.ccp.uima.annotation.CCPDocumentInformation");
127        if (lowLevelTypeChecks)
128          ll_cas.ll_setStringArrayValue(ll_cas.ll_getRefValue(addr, casFeatCode_secondaryDocumentIDs), i, v, true);
129        jcas.checkArrayBounds(ll_cas.ll_getRefValue(addr, casFeatCode_secondaryDocumentIDs), i);
130        ll_cas.ll_setStringArrayValue(ll_cas.ll_getRefValue(addr, casFeatCode_secondaryDocumentIDs), i, v);
131      }
132     
133     
134      /** @generated */
135      final Feature casFeat_classificationType;
136      /** @generated */
137      final int     casFeatCode_classificationType;
138      /** @generated */ 
139      public String getClassificationType(int addr) {
140            if (featOkTst && casFeat_classificationType == null)
141          jcas.throwFeatMissing("classificationType", "edu.uchsc.ccp.uima.annotation.CCPDocumentInformation");
142        return ll_cas.ll_getStringValue(addr, casFeatCode_classificationType);
143      }
144      /** @generated */    
145      public void setClassificationType(int addr, String v) {
146            if (featOkTst && casFeat_classificationType == null)
147          jcas.throwFeatMissing("classificationType", "edu.uchsc.ccp.uima.annotation.CCPDocumentInformation");
148        ll_cas.ll_setStringValue(addr, casFeatCode_classificationType, v);}
149        
150      
151    
152    
153    
154      /** initialize variables to correspond with Cas Type and Features
155            * @generated */
156      public CCPDocumentInformation_Type(JCas jcas, Type casType) {
157        super(jcas, casType);
158        casImpl.getFSClassRegistry().addGeneratorForType((TypeImpl)this.casType, getFSGenerator());
159    
160     
161        casFeat_documentID = jcas.getRequiredFeatureDE(casType, "documentID", "uima.cas.String", featOkTst);
162        casFeatCode_documentID  = (null == casFeat_documentID) ? JCas.INVALID_FEATURE_CODE : ((FeatureImpl)casFeat_documentID).getCode();
163    
164     
165        casFeat_documentCollectionID = jcas.getRequiredFeatureDE(casType, "documentCollectionID", "uima.cas.Integer", featOkTst);
166        casFeatCode_documentCollectionID  = (null == casFeat_documentCollectionID) ? JCas.INVALID_FEATURE_CODE : ((FeatureImpl)casFeat_documentCollectionID).getCode();
167    
168     
169        casFeat_documentSize = jcas.getRequiredFeatureDE(casType, "documentSize", "uima.cas.Integer", featOkTst);
170        casFeatCode_documentSize  = (null == casFeat_documentSize) ? JCas.INVALID_FEATURE_CODE : ((FeatureImpl)casFeat_documentSize).getCode();
171    
172     
173        casFeat_secondaryDocumentIDs = jcas.getRequiredFeatureDE(casType, "secondaryDocumentIDs", "uima.cas.StringArray", featOkTst);
174        casFeatCode_secondaryDocumentIDs  = (null == casFeat_secondaryDocumentIDs) ? JCas.INVALID_FEATURE_CODE : ((FeatureImpl)casFeat_secondaryDocumentIDs).getCode();
175    
176     
177        casFeat_classificationType = jcas.getRequiredFeatureDE(casType, "classificationType", "uima.cas.String", featOkTst);
178        casFeatCode_classificationType  = (null == casFeat_classificationType) ? JCas.INVALID_FEATURE_CODE : ((FeatureImpl)casFeat_classificationType).getCode();
179    
180      }
181    }
182    
183    
184    
185
```
